# Re-Tubularization of Highly-Ischemic Anti-Mesenteric Border (ReHAB): A Novel Bowel Preservation Technique in Complex Gastroschisis

**DOI:** 10.21699/jns.v6i3.602

**Published:** 2017-08-10

**Authors:** Richard J. Hendrickson, Ashwini S. Poola, Katherine W. Gonzalez, Joel Lim, Tolulope A. Oyetunji

**Affiliations:** 1 Department of Pediatric Surgery, Children’s Mercy Hospital; 2 Department of Pediatric Gastroenterology, Children’s Mercy Hospital

**Keywords:** Gastroschisis, Ischemic bowel, Short bowel syndrome

## Abstract

Complex gastroschisis with bowel necrosis poses an operative challenge. Surgeons must weigh the decision between resection versus preservation of ischemic bowel. As one of the leading causes of short bowel syndrome, aggressive resection in complicated gastroschisis subjects children to prolonged dependence on parenteral nutrition and its attendant complications. Herein, we describe a novel technique aimed towards bowel preservation in complex gastroschisis patients with severe bowel ischemia with the ultimate goal for enteral autonomy.

## INTRODUCTION

Gastroschisis is the most common abdominal wall birth defect with increasing incidence worldwide [1]. While outcomes for simple cases are quite favorable, complicated gastroschisis pose significant operative challenges. Aggressive surgical resection often results in short bowel syndrome (SBS) and intestinal failure [2]. A recent analysis showed that children with intestinal failure on home parenteral nutrition (HPN) incur a mean cost of $320,000 per child in the first year post-discharge. [3] Therefore, methods to achieve enteral autonomy not only limits costs but improves the overall quality of life for these patients and their caregivers. We propose a novel surgical technique that preserves native bowel while ultimately achieving enteral autonomy with little alteration of natural intestinal physiology—targeting resection towards the predictable area of ischemia: the anti-mesenteric border.

## CASE SERIES

**Case 1:**

Caucasian male with prenatally detected gastroschisis was born at 34 weeks gestation to a G3P1 mother via induced vaginal delivery due to non-reassuring fetal status. Initial management included gastric decompression, endotracheal intubation and fluid resuscitation. A preformed silastic spring-loaded silo (Specialty Surgical Products, Inc, Montana, US) was placed at the bedside for graduated daily manual reduction of the eviscerated bowel. The intestine was noted to have patchy segments of ischemia along the anti-mesenteric border. Due to persistent metabolic acidosis despite resuscitation, the patient was taken to the operating room (OR) for concerns of bowel necrosis. Intra-operatively, a long segment of small intestine was noted to be ischemic with frank necrosis along the anti-mesenteric border approximately 15 cm proximal to the ileocecal valve (ICV) as identified by the appendix. The estimated bowel length proximal to the necrosis was approximately 15 cm to the presumed duodenum. Due to the extensive edema and matted intestine along with a foreshortened mesentery, the exact length of intestine from the duodenum to ICV was difficult to measure.

The decision was made to excise the necrotic anti-mesenteric segment and “re-tubularize” or “ReHAB” this segment. A longitudinal incision with electrocautery was made along the entire anti-mesenteric length of the highly ischemic and necrotic bowel. This was then fully excised being careful not to compromise the bowel near the mesenteric border. A 16 French catheter was placed within the lumen and the bowel was closed over the catheter, thereby re-tubularizing this segment with a running 5-0 PDS suture from the midpoint to the either end of excision (Fig.1). The suture line was tested with saline and interrupted sutures were placed to reinforce any areas of leakage. A silo was replaced to enable daily examination of the bowel.

**Figure:**
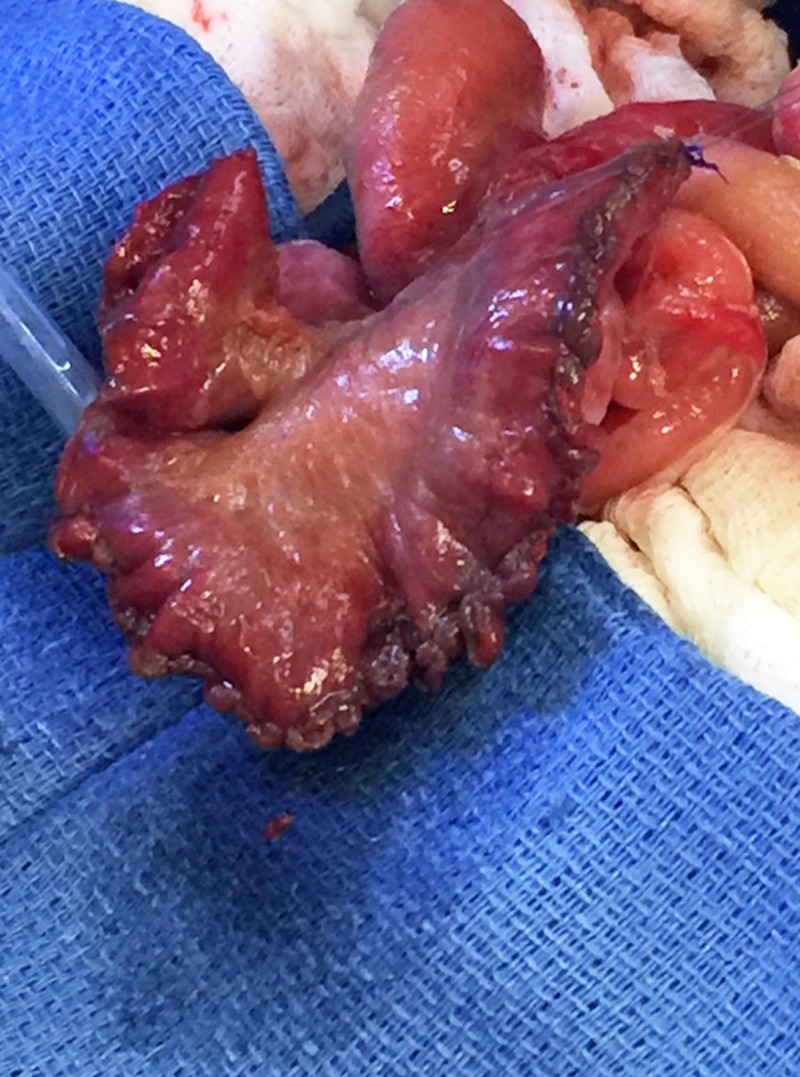
Figure 1. Targeted anti-mesenteric resection of necrotic bowel and tubularization over 16Fr catheter.

Manual reduction of bowel in the silo was started on post-operative day (POD) 5. The patient returned to the OR for abdominal wall closure once the bowel was fully reduced on POD 8. The “ReHAB” intestine appeared viable, the suture line was buttressed and, a jejunostomy and mucous fistula were created proximal to the “ReHAB” intestine to protect the repair. The fascia was closed with stomas brought out laterally. Ostomy started moving 7 days after closure. A contrast study via the mucous fistula to evaluate for refeeding the distal segment revealed a leak and the bowel was left in discontinuity for six weeks. Upon return to the OR for re-anastomosis, a previously unrecognized ileal atresia, the source of the prior leak, was identified and resected distal to the “ReHAB” segment. A 3 cm portion of the “ReHAB” bowel was resected along with the atretic portion. Intestinal continuity was established. A total of 90 cm of healthy small intestine in continuity from the duodenum to ICV remained at the end of this case. The bowel was easily reduced and the abdominal wall was closed without difficulty.

Enteral feeds were steadily advanced to full volume caloric requirement over 9 days. Full enteral autonomy was achieved before discharge with no requirement for HPN. The patient continues to do well with full enteral autonomy, and is followed routinely as an outpatient by the Intestinal Rehabilitation Team.

**Case 2:**

Caucasian male with prenatally detected gastroschisis was born at 37 weeks gestation to a G1P1 mother via cesarean section. Similar to case 1, initial management included bedside silo placement, intubation and fluid resuscitation. The intestine appeared dusky and darker in coloration but no frank necrosis was noted. Over the first 24 hours, the patient remained hemodynamically stable without signs of worsening sepsis. However, the bowel appeared blackened with significant concern for frank necrosis. In the OR, there was significant ischemia to the colon, appendix, small bowel, and stomach due to torsion. A 17 cm portion of jejunum was black and nearly necrotic along the antimesenteric aspect. The bowel was detorsed and improvement in perfusion was noted; silo was replaced. Due to concerns for worsening ischemia, the patient returned to the OR on POD 3. Using electrocautery a longitudinal incision was made at the anti-mesenteric border and this necrotic portion was excised. A ReHAB segment was created as previously described.

On day of life 12, the patient returned to the OR for abdominal closure. Due to bowel wall edema and lack of adequate fascia for a tension free closure, a Permacol patch (Covidien Surgical, Mansfield, MA US) was placed as an onlay over the fascial defect. Skin was closed over the patch. Free intra-peritoneal air was noted on serial radiograph and he returned to the OR on POD 3. There were two small areas of perforation at the proximal and distal end of “ReHAB” segment of intestine. The distal perforation was repaired primarily; however, the proximal perforation was too friable for repair and was ultimately resected. Surgical drains were placed and removed three weeks later. His post-operative course was complicated by a surgical site infection requiring parenteral antibiotics.

Due to failure to advance on oral feeds, the patient underwent exploration with adhesiolysis, Ladd’s procedure and gastrostomy tube placement. The abdomen was closed primarily. Bowel movements were noted the very next day and consistently continued from then on. The patient was discharged home at 6 months of age on full enteral feeds and has not required any additional surgical interventions. The patient continues to do well with full enteral autonomy, and is followed routinely as an outpatient by the Intestinal Rehabilitation Team.

## DISCUSSION

We describe a technique which favors intestinal rehabilitation in case of extensive ischemic bowel. The first step is intra-operative decision making. When there is compromised bowel pathology, e.g. ischemia or necrosis, the key decision is to resect or not. The paramount concern for resection is the potential for SBS. While there is no strict anatomic definition for SBS, longer residual bowel has been associated with an improved chance of achieving enteral autonomy in SBS patients [4]. This is the basis for operative techniques, like the Bianchi or STEP procedures, in the management of SBS to lengthen the intestine [5,6]. In our case, complete resection of the necrotic intestine would have left the patients with significantly reduced intestinal length. Resecting just the pathologic anti-mesenteric segment of bowel left the native “length” of intestine intact with the hope of allowing this “ReHAB” segment to have suitable capacity for absorption and future growth.

This principle has only been reported once before as a salvage technique in a gastroschisis infant with significant midgut volvulus and ischemia [7]. Our technique differs because the highly ischemic antimesenteric segment was excised without compromising intestinal continuity on the mesenteric side. There is room for improvement in this procedure. Both patients developed leaks, which were clinical setbacks in their overall clinical course for eventual enteral autonomy. A protective ostomy will be a good option for our future cases. Additionally, buttressing the repair with interrupted sutures, while being careful not to constrict the lumen, may strengthen the repair on compromised tissues.

Our patients maintained adequate enteral autonomy with adequate weight gain with no need for HPN. Long term outcomes are imperative in determining the continued nutritional status, growth and development of these patients; we feel hopeful that this will be considered a viable and easily reproducible form of management in complex gastroschisis patients and other patients prone to intestinal failure and SBS.

## Footnotes

**Source of Support:** None

**Conflict of Interest:** None

## References

[1] Centers for Disease Control and Prevention (CDC). Hospital stays, hospital charges, and in-hospital deaths among infants with selected birth defects--United States, 2003. MMWR Morb Mortal Wkly Rep. 2007;56:25–9. 17230142

[2] D’Antonio F, Virgone C, Rizzo G, Khalil A, Baud D, Cohen-Overbeek TE, et al. Prenatal risk factors and outcomes in gastroschisis: A meta-analysis. Pediatrics. 2015;136:e159–69. 10.1542/peds.2015-0017 26122809

[3] Kosar C, Steinberg K, de Silva N, Avitzur Y, Wales PW. Cost of ambulatory care for the pediatric intestinal failure patient: One-year follow-up after primary discharge. J Pediatr Surg. 2016; 51:798-803. 10.1016/j.jpedsurg.2016.02.026 26932248

[4] Khan FA, Squires RH, Litman HJ, Balint J, Carter BA, Fisher JG, et al. Predictors of enteral autonomy in children with intestinal failure: A multicenter cohort study. J Pediatr. 2015;167:29–34.e1. 10.1016/j.jpeds.2015.03.040 PMC4485931 25917765

[5] Bianchi A. Intestinal loop lengthening—A technique for increasing small intestinal length. J Pediatr Surg. 1980;15:145–51. 10.1016/s0022-3468(80)80005-4 7373489

[6] Kim HB, Fauza D, Garza J, Oh J-T, Nurko S, Jaksic T. Serial transverse enteroplasty (STEP): A novel bowel lengthening procedure. J Pediatr Surg. 2003;38:425–9. 10.1053/jpsu.2003.50073 12632361

[7] McCullagh M, Garvie DC, Dykes EH. Papers Presented at the 25th Annual Meeting of the Canadian Association of Paediatric Surgeons. A new method of intestinal salvage for severe small bowel ischemia. J Pediatr Surg. 1994;29:1231–3. 10.1016/0022-3468(94)90809-5 7807353

